# Acute and Lifetime Stress and Psychotic Illness: The Roles of Reward and Salience Networks

**DOI:** 10.20900/jpbs.20220012

**Published:** 2022-12-30

**Authors:** Jacob L. Nudelman, James A. Waltz

**Affiliations:** Maryland Psychiatric Research Center (MPRC), Department of Psychiatry, University of Maryland School of Medicine, Baltimore, MD 21228, USA

**Keywords:** affective reactivity, sensitization, dopamine, serotonin, prediction error, reinforcement learning, striatum, insula

## Abstract

Affective reactions to acute stressors often evoke exacerbations of psychotic symptoms and sometimes de novo psychotic symptoms and initial psychotic episodes. Across the lifespan, affective reactions to acute stressors are enhanced by successive adverse childhood experiences (ACEs), in a process called “behavioral sensitization”. The net effects of behavioral sensitization of acute stress responses are to alter responsivity to positive and negative feedback and to unexpected events, regardless of valence, leading to the maladaptive assignment of salience to stimuli and events. The assignment of “aberrant” salience to stimuli and events has profound consequences for learning and decision-making, which can influence both the positive and negative symptoms of psychosis. In this review, we discuss some of the psychological and neural mechanisms by which affective reactivity to acute stress, and its sensitization through the experience of stress and trauma across the lifespan, impact both the positive and negative symptoms of psychosis. We recount how the reward and salience networks of the brain, together with inputs from the dopamine and serotonin neurotransmitter systems, are implicated in both affective reactivity to stress and the symptoms of psychosis, likely mediate the effects of stress and trauma on the symptoms of psychosis and could serve as targets for interventions.

## INTRODUCTION

Evidence indicates that acutely stressful experiences are often followed by exacerbations of psychotic symptoms, in vulnerable populations, and sometimes by de novo psychotic symptoms [1–3]. An ability of affective reactivity to daily-life stressors to engender and exacerbate psychotic symptoms, in a variety of populations, is suggested by the results of numerous studies, using multiple techniques, including the ecological momentary assessment (EMA) method [4–9] and the induction of acute stress [10], using paradigms such as the Montreal Imaging Stress Task [11,12].

In the context of experimental tasks, acute stress is induced by painful physical stimuli, such as extreme heat or cold [13] or electric shock [14], psychosocial conditions, such as criticism or unsympathetic expressions from peers [12,15], or a combination of the two [16,17]. The severity of perceived stress is often operationalized according to elevated cortisol levels [18], electrodermal responses [19], increased heart-rate or heart-rate variability [20], and ratings of perceived stress on self-report instruments [21]. Acute stress can significantly impact what the brain deems to be “salient” [22], and has been shown to enhance associative learning [23]. The “aberrant salience” hypothesis of psychosis posits that, in individuals with psychotic illness, the brain assigns salience to normally-mundane stimuli, leading to odd perceptions and experiences, requiring explanation [24,25]. The interpretations of these odd perceptions and experiences are thought to lead to the emergence of unusual beliefs (delusions) [25–28].

The term “adverse childhood events” (ACEs) encompasses a wide range of chronic stressors including abuse, neglect, lower socioeconomic status, urbanicity, family instability, and other such experiences. These chronic stressors are generally associated with higher levels of psychopathology, although the precise mechanisms by which this occurs are debated. The repeated or chronic experience of ACEs over time can enhance affective reactivity to acute stressors, in a process called “behavioral sensitization” [29].

The purpose of this review is to connect several concepts related to the affective reactivity hypothesis of psychosis and to discuss potential mechanisms by which ACEs can contribute to psychosis in adulthood. In particular, we will discuss (1) how ACEs are thought to sensitize dopamine systems, thereby increasing reactivity to acute stressors and the positive symptoms of psychosis; and (2) how ACEs are thought to impact hedonics and motivation, thereby increasing the negative symptoms of psychosis. We will consider the question of whether effects of ACEs on positive and negative symptoms of SZ are connected or separate.

## NEURAL AND BEHAVIORAL CORRELATES OF AFFECTIVE REACTIVITY TO ACUTE STRESSORS

**Figure 1.**
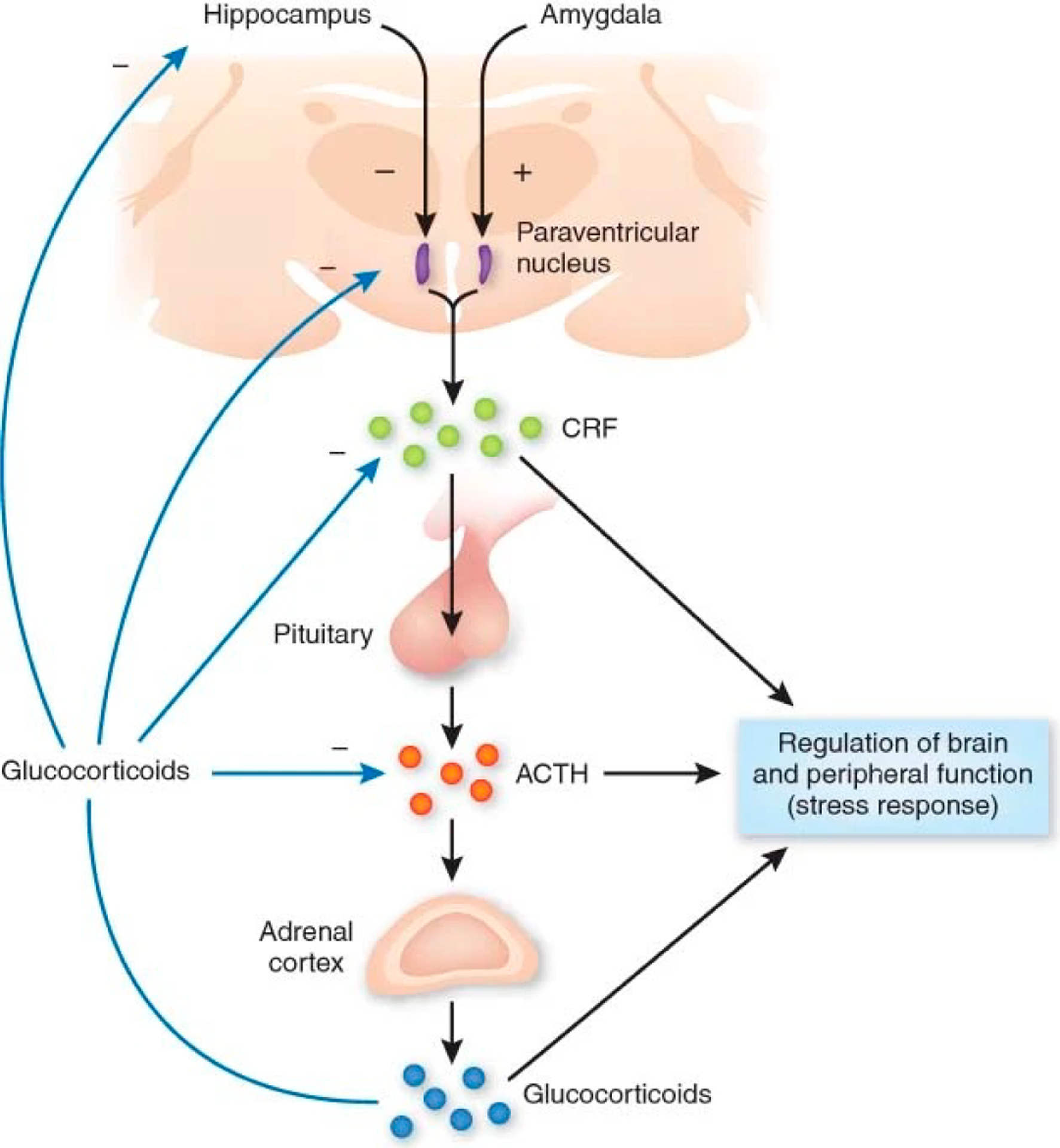
The hypothalamic-pituitary-adrenal (HPA) axis. The release of corticotropin-releasing factor (CRF) by the hypothalamus promotes release of adrenocorticotrophic hormone (ACTH) by the pituitary gland, which, in turn, signals the adrenal glands to begin releasing glucocorticoids into the blood. Glucocorticoids (such as cortisol) travel via the bloodstream and attach to glucocorticoid receptors in the brain. The hippocampus and amygdala can, in turn, influence the activity of the hypothalamus. Adapted from Hyman [56], with permission copyright ©2009 Springer Nature.

**Figure 2.**
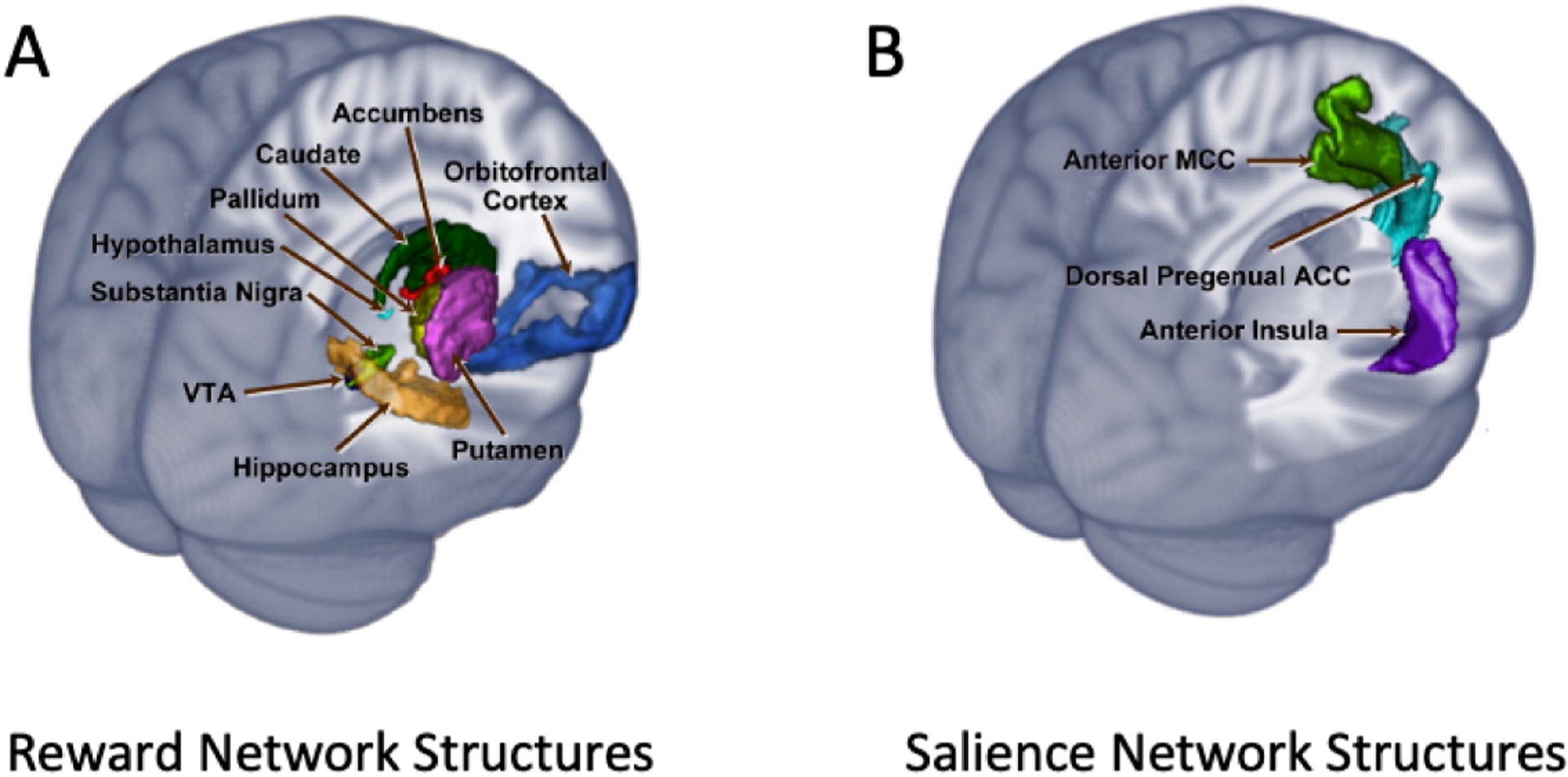
Nodes of the Salience and Reward Networks. (**A**) Salience network: anterior insula, dorsal pregenual anterior cingulate cortex (dorsal pgACC), anterior mid cingulate cortex (aMCC). Reward network: hypothalamus, orbitofrontal cortex (OFC), the ventral striatum (VS), including the nucleus accumbens and ventral putamen, ventral tegmental area (VTA), substantia nigra, midbrain regions (caudate, pallidum). As Haber and Knutson (2010) have noted, other structures including the amygdala, hippocampus, lateral habenular (LHb) nucleus, and brainstem structures, such as the pedunculopontine nucleus and the raphe nuclei, play key roles in regulating the reward network. (**B**) Adapted from Gupta et al. [57], with permission copyright © 2015 Elsevier.

Affective reactivity to acute stressors recruits numerous neural and endocrine systems, with widespread downstream effects [30,31]. Specifically, stress-induced activity of the hypothalamic-pituitary-adrenal (HPA) axis (Figure 1) and the release of glucocorticoids from the adrenal cortex are thought to evoke activation of brain dopamine (DA) [10,32–34] and serotonin systems (5-hydroxytryptamine, or 5HT) [35–37]. Beyond dopamine and serotonin circuits, affective reactivity to acute stressors recruits limbic circuits, as well, implicating the hippocampi, anterior insula (AI), anterior and posterior cingulate cortices (ACC/PCC), precuneus, and supramarginal gyrus (SMG) [38–40]. Many of these regions comprise nodes of the “Salience Network” (Figure 2A) [41], which has also been closely linked to psychosis [42–44].

Acute stress has a particular influence on activity in reward circuits (Figure 2B), by virtue of its effects on brain dopamine systems (VS) [45,46]. These alterations of reward system function have important consequences for learning and behavior. For example, acute stress has been associated with attenuated reward responsiveness [47–49]. Specifically, dopamine neurons and their targets in reward circuits have been shown to play an essential role in attribution of salience to stimuli and events [50,51], and, in particular, in the signaling of reward prediction errors (RPEs)—a kind of salient event critical to reinforcement learning (RL) [52,53]. Acute stress has been shown to increase sensitivity to negative prediction errors, relative to positive prediction errors [47,54]. The blunting of *positive* RPEs and enhancement of *negative* RPEs would have profound consequences for learning and the subsequent ability of the same stimuli to motivate behavior. Because RPE signaling influences the attribution of incentive value to stimuli [55], altered RPE signaling could result in a reduced ability to adaptively attribute motivational salience to biologically-important stimuli.

## NEURAL CONSEQUENCES OF ACES

When stress accumulates during development, there are profound effects on neural systems [58]. After repeated exposure to highly stressful events, many studies show sensitization of the HPA axis, with the body releasing more cortisol in response to acute stress [59,60]. The chronic activation of the HPA axis is known to lead to increased production of corticotropin releasing factor (CRF) [61], with clear effects on dopamine and serotonin systems [29,62], often, but not always, leading to greater synthesis and release of DA (and 5HT) [10,33–37,62–66]. These effects on neurotransmitter systems are known to have important downstream effects in nodes of the salience [67–71] and reward networks [72–77]. For example, alterations in dopamine signaling may lead to excess noise in frontostriatal circuits [27,78]. Accumulated stress may have the ability to disrupt phasic dopamine/reward signals by virtue of their effects on dopamine tone. Importantly, chronic stress may have opposite effects on dopamine concentrations and receptor function in the striatum and PFC [79]. That is, findings indicate that stress-induced elevations in DA release are often associated with decreased responses to rewards in the PFC [80,81]. While the direction of causality is not clear, there is evidence that hypofrontality may dysregulate DA transmission in the striatum [82]. Finally, recent findings point to disrupted connectivity between PFC and striatum consequent to acute and accumulated stress [83–85].

## LIFETIME STRESS AND BEHAVIORAL SENSITIZATION

High numbers of ACEs have been associated with the emergence of diagnosable clinical disorders of anxiety and mood [86,87]. According to the “behavioral sensitization” hypothesis, the accumulation of chronic stressful experiences during childhood and adolescence can eventually lead to the emergence or exacerbation of psychotic symptoms, as well [29], especially in the case of those with, or at risk for, psychotic disorders [88–90]. In the “behavioral sensitization” framework, the repeated experience of ACEs can make acute stressors more salient, leading to a more pronounced response to acute stressors. This is largely in contrast to the effects of typical stressors during early life, which in some cases is linked to beneficial effects on mental health, including through positive impacts on cognitive performance, motivation, and resilience. These stressors are often more manageable and temporary in nature and can include experiences such as exercise or studying for a test [91].

**Figure 3.**
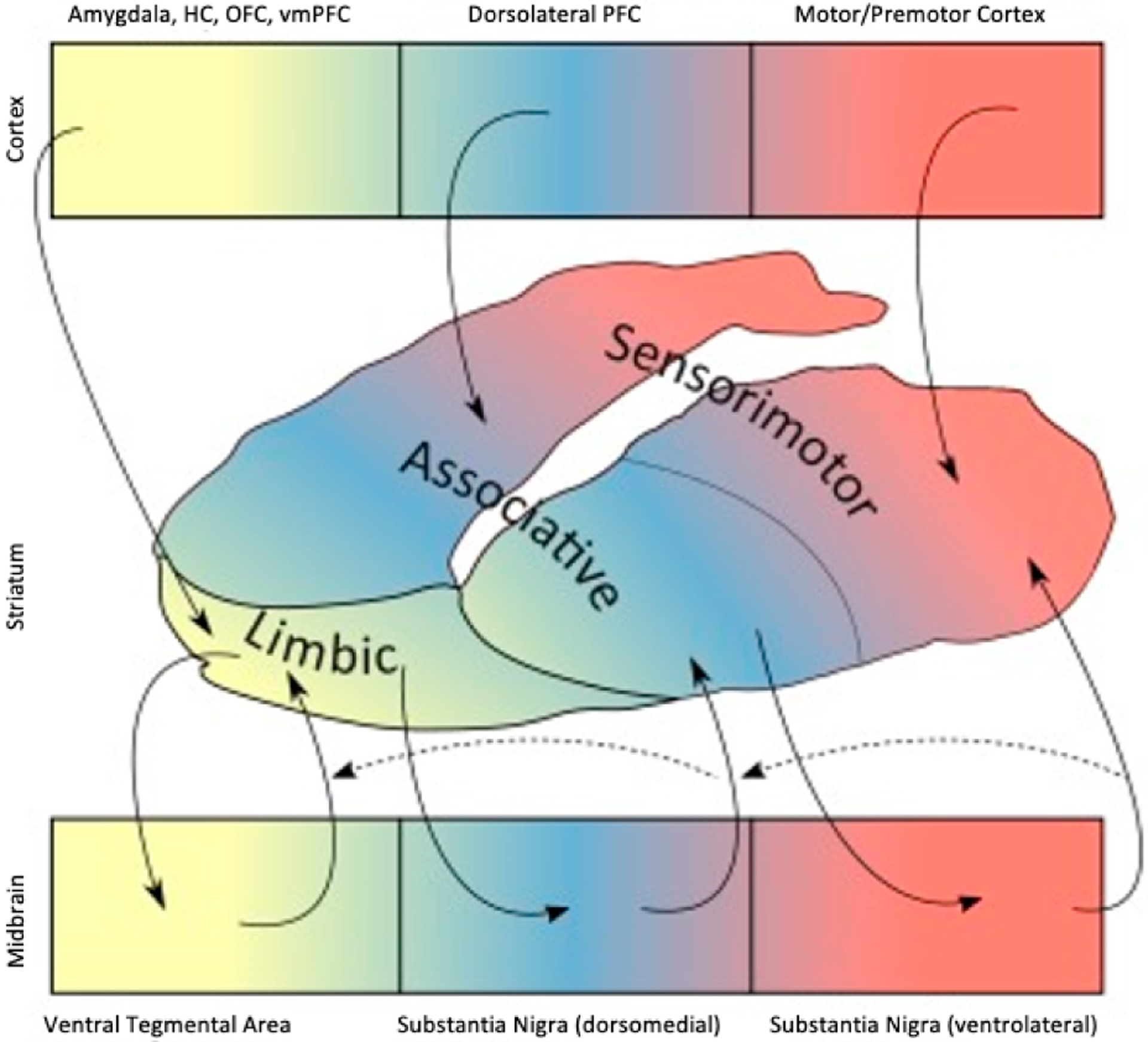
Tract tracing studies have shown that striatocortical connections run in three parallel pathways. Motor areas project to the caudal putamen; dorsolateral prefrontal cortex to caudate and rostral putamen; and limbic areas to the ventral (limbic) striatum. These subdivisions have been termed the sensorimotor, associative, and ventral (limbic) striatum. The ventral tegmental area and medial substantia nigra (SN) project primarily to limbic striatum, while central/ventrolateral parts of the SN project to the associative and sensorimotor striatum. Striatal efferents projecting back to the midbrain. In addition to these reciprocal connections, feedforward striato-nigro-striatal connections allow information to pass along the striatum from limbic to motor regions via the associative striatum. Adapted from McCutcheon et al. [27], an open access article distributed under the Creative Commons Attribution 4.0 International License (http://creativecommons.org/licenses/by/4.0/).

Due to their involvement in both stress reactivity and the positive symptoms of psychosis, brain DA systems, and their targets in the reward network have long been thought to *mediate* the relationship between acute stress reactivity and the symptoms of psychosis [5,29,90,92,93]. In recent years, there has been an additional focus on the associative striatum (AS), located lateral to the limbic and sensorimotor striatum (Figure 3). There is evidence that, in individuals along the psychosis spectrum, the associative striatum is the striatal region where chronic stress has its greatest impact on psychosis severity, by virtue of sensitizing dopamine systems [10,94]. While there is evidence that the AS may show responses to acute stressors reflective of behavioral sensitization in people along the psychosis spectrum [10], the relationship between elevated DA activity in the AS and attenuated reward signals in the ventral striatum (VS) is not clear. These phenomena may even occur relatively independently, with elevated dopamine synthesis capacity linked primarily to positive symptoms, and blunted reward signaling most closely tied to negative symptoms [95].

## BEHAVIORAL CONSEQUENCES OF DOPAMINE SYSTEM SENSITIZATION: ABERRANT SALIENCE SIGNALING

What are the exact neural mechanisms by which accumulated stress could exacerbate psychotic symptoms? As noted above, elevated reactivity to acute stress is thought to be accompanied by increased noise (reduced reliability) in frontostriatal circuits [27,78], which may lead to the assignment of both too much and too little salience to stimuli and events, depending on the particular situation. That is, behavioral sensitization may result in an elevated baseline, against which reward-related phasic dopamine signals may be difficult to interpret.

Beyond enhancing reactivity to acute stressors, dopamine system alterations resulting from accumulated stress may bring about a more general disruption of the ability to adaptively assign salience to external stimuli and events, leading to, for example, alterations in the signaling of negative and positive prediction errors and one’s sensitivity to rewards and punishments. They may also lead to an increased tendency to associate mundane stimuli with negative valence. Of note, unmedicated psychotic illness is associated with both elevated dopamine tone [96,97] and attenuated RPE signaling [95,98]. Specific associations between ACEs and reward-related responses in the VS have been observed in multiple studies [77,99]. Associations between ACEs and nonrewarding salience signals have been observed in insula and amygdala [100–102]. These alterations in reward and salience signals have been specifically linked to psychopathology in individuals with depression [74,76] and in adolescents and young adults at clinical high-risk for psychosis [103].

Since the aberrant salience framework first emerged, numerous studies have been conducted to investigate how the aberrant signaling of salience might mediate the relationship between dopamine system sensitization and psychotic symptoms [104–108]. In a neuroimaging study, McCutcheon and colleagues [109] established a direct link between corticostriatal connectivity and multiple behavioral measures of salience perception. Specifically, these researchers found that, in a sample with a high degree of exposure to chronic psychosocial stressors, the strength of connectivity between the VS and brain regions implicated in salience processing negatively correlated with explicit adaptive salience and positively correlated with aberrant salience measures [109].

Several other factors are likely to determine the impact of accumulated stress on the severity of psychotic symptoms. First, the behavioral consequences of ACEs are likely to differ as a function of which frontostriatal loops are most affected [27]. For example, ACEs impacting the limbic/ventral striatum might be more likely to affect reward processing and value-based decision-making also involving ventromedial prefrontal cortex (vmPFC), whereas ACEs impacting dorsolateral striatum might be more likely to affect cognitive control processes involving dorsolateral prefrontal cortex (dlPFC). Second, it is conceivable that disrupted connectivity between PFC and striatum consequent to accumulated stress [83–85] results in a reduced ability to use striatal salience/PE signals to update value representations in vmPFC and/or use volatility in the environment to modulate attention to feedback. In this way, the neural effects of ACEs could lead to both impairments in RL and an increased tendency to signal salience in an aberrant manner. Third, excessive salience attribution might also result in a general disengagement from some reward and salience processes as resources are diverted to regions and networks related to stress and negative affect [85]. In the case of psychotic illness, it is essential to remember that the effects of accumulated stress occur against a background of genetic vulnerabilities and/or disease processes, and thus may interact with and accentuate elevations of dopamine tone consequent to these vulnerabilities and/or processes.

## DIMENSIONS OF TRAUMA, DIMENSIONS OF SYMPTOMS

It is also important to note that, although early life experiences of chronic stress in all forms can be detrimental to mental health, research shows that not all types of traumatic experiences are equally involved in the development of behavioral sensitization and exacerbations in psychotic symptoms. Measures of childhood trauma, such as the Adverse Childhood Events Scale [110] and the Childhood Trauma Questionnaire (CTQ) [111], are often separated into various domains based on the type of stress experienced. Most of these domains can be further separated into two main dimensions—one involving direct experiences of threat or violence (e.g., sexual, physical, and emotional abuse), and another involving a deficit in basic necessities for healthy development (e.g., physical and emotional neglect, poverty) [112–114]. This separation is notable because these two groups of ACEs seem to differentially impact the development of cognitive, emotional, and neural processes, with experiences of threat and violence having a more substantial impact on emotional regulation, while depravation through neglect and poverty has a greater impact on mechanisms of cognitive control [114].

These data suggest that, while the number of ACEs experienced can be informative, it is important to also account for type of adverse event experienced when evaluating risk for the development or worsening of psychotic symptoms. It appears that experiences of threat, in particular, may be more instrumental in the development of affective reactivity and behavioral sensitization than other forms traumatic events through deficits in emotional regulation, suggesting a greater impact on positive psychotic symptoms [115,116].

## EFFECTS OF ACUTE AND LIFETIME STRESS ON NEGATIVE SYMPTOMS.

While much work has focused on the contributions of stress to the positive symptoms of psychosis [5,29,90,92,93], as well as depression [86,87], considerably less work has devoted to the investigation of the potential contributions of stress to *negative* symptoms in psychotic illness, like anhedonia and motivational deficits (avolition). Nonetheless, it is well-established that the profound neurobiological alterations associated with cumulative stress lead not only to increased *psychotic* reactivity to stress, but *also* play a role in the development and exacerbation of negative symptoms [117,118].

There are several potential mechanisms by which adverse childhood experiences could contribute to anhedonia, avolition, and other negative symptoms of psychotic illness. First, as noted above, there is evidence that different kinds of ACEs might impact dopamine and serotonin systems differently, and, consequently, future sensitivity to stressors, punishments, and rewards. It is important to note that the chronic activation of the HPA axis doesn’t always lead to greater synthesis and release of DA; under some conditions, chronic stress engenders the suppression of DA activity [66,119–121]. Studies in rodents have demonstrated increased anhedonic behaviors after maternal neglect [61], and ACEs have been shown to *suppress* reward system activity in the human brain, thereby altering the assignment of incentive salience to stimuli, in some studies with human subjects [74,75,77,122]. In addition to being associated with reduced reward sensitivity, the accumulation of ACEs has been shown to have an effect similar to individual acute stressors in heightening punishment sensitivity [123,124]. There is clear evidence that negative symptoms like anhedonia and avolition have been associated with both blunted reward responsiveness/RL [125–127] and reduced activity in the same frontostriatal circuits impacted by stressful and traumatic events across the lifespan [128–134].

Second, there is strong evidence that different kinds of ACEs might impact various emotional and cognitive processes in disparate ways. For example, depravation through neglect and poverty has been shown to have a greater impact on mechanisms of cognitive control than on emotional reactivity [112–114], and child abuse and neglect have been associated with distinct patterns of performance on emotion discrimination tasks [135]. Given these observations, it is not surprising that depravation has been found to have similar effects on processes related to motivation and pleasure, in psychotic illness [115].

Thus, ACEs related to abuse appear to contribute to the negative symptoms of psychosis by increasing sensitivity to punishments relative to rewards, whereas ACEs related to neglect appear to contribute to the negative symptoms of psychosis by having a detrimental impact on motivation, learning, and cognition. That is, while dopamine sensitization and increased affective reactivity to acute stress may contribute to both the positive and negative symptoms of psychosis, negative symptoms such as anhedonia and avolition may be influenced by additional factors unrelated to dopamine sensitization. We contend that understanding the potential links between chronic stress and negative symptoms is vital, due to the contributions of negative symptoms to real-world functioning in psychotic illness [136,137], as well as the paucity of effective treatments for them [138,139].

## INTERPLAY BETWEEN REWARD AND SALIENCE NETWORKS

Given that motivational, or incentive, salience [55,140] is an important form of salience, it is not surprising that most, if not all, neural systems implicated in salience signaling subserve motivational processes, as well. It is also important to note that brain networks for acute stress reactivity, salience signaling, and reward sensitivity are separable, but overlapping, with the implication that the function being performed by a region likely depends on the network in which it is participating. For example, VS has been implicated in both reward processing *and* in the signaling of salient events, regardless of valence [141–143], and thus appears to be a node shared by both Reward and Salience Networks. Serotonin systems also play a role in both feedback processing and in the signaling of salient events [144–146], likely due to their projections to the amygdalae and other Salience Network nodes [41,147,148]. Finally, there is evidence that both the Reward and Salience Networks figure critically in reinforcement learning by signaling signed and unsigned prediction errors, respectively, with the precision of prediction errors possibly influencing rates of prediction-error-driven learning [108,149,150]. Our group has shown that disrupted unsigned prediction error signals in prefrontal cortex relates to motivational deficits in schizophrenia patients [151].

## CONCLUSIONS

**Figure 4.**
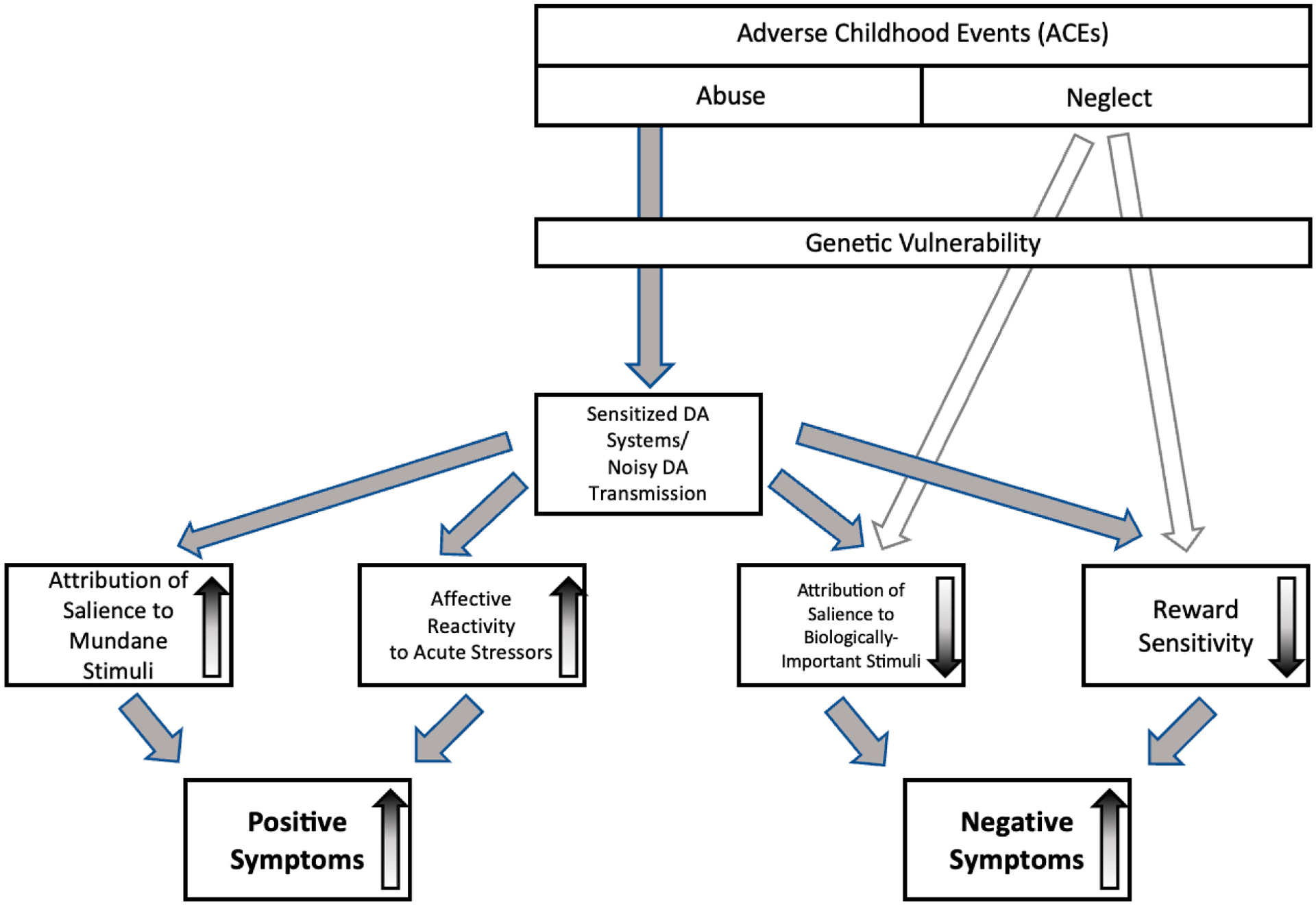
Adverse Childhood Experiences (ACEs) act through sensitization of dopamine systems to impact sensitivity to stressors, punishments, and rewards and the attribution of salience to events (which range from mundane to biologically-important). Conversion to psychotic illness and the expression of psychotic symptoms are influenced by affective reactivity to stimuli and events and the attribution of salience to these stimuli and events. Negative symptoms may emerge as a consequence of noisy dopamine signaling, if (1) people attribute insufficient motivational salience to biologically-important stimuli and events, or if (2) reductions in prefrontal cortical activity levels are associated with striatal hyperactivity, leading to deficits in reward sensitivity, motivation, and decision-making. Some negative symptoms in psychotic illness may result from mechanisms separate from sensitization of dopamine systems, as growing evidence suggests that negative symptoms are more closely tied to neglect and deprivation in childhood/adolescent, whereas positive symptoms are more closely tied to abuse.

The findings reported above highlight the importance of understanding the roles of frontostriatal circuits in assigning salience to stimuli and events, as well as the potential value of using precise behavioral and neural measures of salience attribution, from experimental paradigms. Based on the findings described above, we can now envision a model of the pathways by which traumatic experiences during childhood and adolescence sensitize susceptible individuals to the noxious effect of future stressors (Figure 4). While these findings *suggest* that dysfunction in salience and reward systems *mediates* relationships between chronic stress and the symptoms of psychosis, direct evidence is scant, and there is a clear need for future investigation into how different dimensions of childhood and adolescent adversity contribute specifically to the link between ACEs and the development of different symptoms of schizophrenia. The reward and salience networks of the brain are likely to serve as important target for intervention, in the development of pharmacological treatments for psychosis, while increasing resilience, regarding affectivity reactivity to stress, should remain a focus for psychological interventions, such as cognitive behavioral therapy.

## Data Availability

Data sharing not applicable to this article as no datasets were generated or analyzed during the current study.
